# Consumption of ultra-processed foods and low dietary diversity are associated with sedentary and unhealthy eating behaviors: A nationwide study with Brazilian Schoolchildren

**DOI:** 10.1371/journal.pone.0294871

**Published:** 2024-01-12

**Authors:** Giovanna Angela Leonel Oliveira, Vivian Siqueira Santos Gonçalves, Eduardo Yoshio Nakano, Natacha Toral

**Affiliations:** 1 Faculty of Health Science, Graduate Program in Human Nutrition, University of Brasilia, Brasilia, Distrito Federal, Brazil; 2 Faculty of Health Science, Graduate Program in Public Health, University of Brasilia, Brasilia, Distrito Federal, Brazil; 3 Department of Statistics, Institute of Exact Sciences, University of Brasilia, Brasilia, Distrito Federal, Brazil; Universidade de Sao Paulo, BRAZIL

## Abstract

**Background:**

Consumption of ultra-processed foods and low dietary diversity are risk factors for chronic diseases.

**Aim:**

To evaluate the association between food consumption and sedentary and unhealthy eating behaviors of Brazilian schoolchildren between 6 and 11 years old.

**Methods:**

Cross-sectional study. A prevalence sample was calculated considering the number of children enrolled in elementary school. This sample was distributed proportionally to Brazil’s macro-regions and the type of school (public or private). The questionnaire was developed in Google Forms and disseminated through the snowball technique. The questionnaire was filled in by the children’s parents, with information about the child’s identification and health. Afterward, the child completed a questionnaire by her/himself. We used the previously validated Illustrated Questionnaire on Food Consumption for Brazilian Schoolchildren and the Illustrated Questionnaire on Eating and Sedentary Behaviors. Food consumption was analyzed using the NOVA score and the dietary diversity score. Poisson’s regression with robust variance was performed (p<0.05).

**Results:**

The study included 2,021 dyads. Of these, 27.6% of children reported eating five or more ultra-processed foods and 39.0% four or fewer natural or staple foods the previous day. Using screens, proxy of sedentary behavior (Prevalence Ratio–PR = 1.8, Confidence Interval–CI_95%_1.2–2.8) and eating at irregular hours (PR = 1.6, CI_95%_1.2–2.2) were risk factors for high consumption of ultra-processed foods and low dietary diversity in schoolchildren. In addition, eating the three main meals on the previous day (PR = 0.6, CI_95%_0.4–0.8) was identified as protective factors against the consumption of ultra-processed foods and in favor of dietary diversity among schoolchildren.

**Conclusion:**

Sedentary and unhealthy eating behaviors were associated with the consumption of ultra-processed foods and low dietary diversity in Brazilian schoolchildren.

## Introduction

Chronic diseases are a burden on the economy and the world’s leading causes of death and loss of quality of life [1]. In 2017, more than 2.1 billion children were affected by chronic diseases, and more than two-thirds of risk factors for chronic diseases emerged during childhood and adolescence [2]. The last Global Burden of Diseases showed that in 2019 non-communicable diseases killed equivalent to 22.5% of children 5–9 years globally and 44.4% in Brazil [3]. Non-communicable diseases, also known as chronic diseases, tend to have slow progress and long duration. They result from a combination of sociodemographic, genetic, physiological, environmental, medical conditions, and behavioral factors. Unhealthy diets and physical inactivity are modifiable behavioral risk factors that significantly contribute to the development of chronic diseases [4].

Dietary habits are formed during childhood and consolidated in adulthood [5]. A report by the Food and Agriculture Organization of the United Nations (FAO) shows the association between ultra-processed food consumption and the risk of various diet-related non-communicable diseases, such overweight/obesity [6]. In Brazil, primary healthcare data from the Food and Nutrition Surveillance System (SISVAN) in 2022 showed that 93% of children between 5 and 9 years of age had consumed ultra-processed foods on the day prior to the consultation. On the other hand, the prevalence of the consumption of natural or staple foods, such as beans, fruits, and vegetables, was 15%, 13%, and 79%, respectively [7].

Furthermore, children’s eating behaviors can be associated with risk factors for their health and habit formation [8]. The Dietary Guidelines for the Brazilian Population present some healthy eating behaviors such as eating regularly and mindfully, eating in appropriate environments, eating in company, and developing, exercising, and sharing culinary skills [9]. Eating behaviors reflect what and how children eat, and they are influenced by children’s eating patterns, taste preferences, appetite, psychosocial factors, as well as the level of physical activity [10].

Sedentary behavior is associated with poor health outcomes such as increased adiposity, poorer cardiometabolic health, and reduced sleep duration [11]. Sedentary behavior includes activities involving small movements during the day, and the most frequent in children is screen use. The WHO Guidelines on Physical Activity and Sedentary Behavior recommend that children should limit the amount of sedentary time, particularly the amount of recreational screen time [12].

So far, no comprehensive national studies have examined food consumption, eating and sedentary behaviors among schoolchildren or their interconnectedness. The association between food consumption and behaviors has already been evaluated in other audiences, such as adults [13,14], adolescents [15–19], university students [20], pregnant women [21], and early childhood children [22–24]. Consequently, our study aims to fill this gap by investigating the association between food consumption—emphasizing on the consumption of ultra-processed foods and dietary diversity—and sedentary and eating behaviors among Brazilian schoolchildren.

## Methods

### Study design

An online cross-sectional study was conducted as part of the Schoolchildren Nutrition Study (*Estudo de Nutrição de Crianças Escolares*–ENUCE in Portuguese). The project was approved by the Research Ethics Committee of the Faculty of Health Sciences at the University of Brasília (number 3,675,033 on October 31, 2019). The responsible and the children agreed to the Informed Consent Form.

### Participants

The study population consisted of children in the elementary school grades, particularly the first to fifth grades, in public and private Brazilian schools.

A sample was calculated considering a population of 14,533,651 children, referring to the number of enrollments in these elementary school grades according to the 2021 National School Census [25]; a confidence level of 95%; a prevalence of ultra-processed food consumption of 89%, data from SISVAN in 2021 [7]; and a relative error of approximately 0.01%, totaling 2,019 children.

This sample value was distributed proportionally to Brazilian macro-regions and type of school (public and private), according to the population of the 2021 National School Census [25]. We considered adequacy 30% for more or less of the minimum calculated sample of each macro-region and type of school (Table 1).

**Table 1:** Sample distribution according to the Brazilian macro-region and type of school. Brazil, 2022.

Variables	Population estimated by School Census, 2021 n (%)	Calculated sample n (%)	Adequacy 30% for more or less n
**Brazil**	14,533,651 (100%)	2,019 (100%)	2,625–1,413
**Macro-Regions**			
North	1,616,919 (11.12)	225 (11.12)	294–158
Northeast	4,132,922 (28.44)	574 (28.44)	746–402
Central-West	1,167,389 (8.03)	162 (8.03)	212–113
Southeast	5,660,515 (38.95)	786 (38.95)	1,022–550
South	1,955,906 (13.46)	272 (13.46)	354–190
**Type of School**			
Public	11,919,578 (82.01)	1,656 (82.01)	2,153–1,159
Private	2,614,073 (17.99)	363 (17.99)	472–254

Compiled by authors.

All children between 6 and 11 years old enrolled in the first to fifth grades of Brazilian elementary schools with internet access were considered eligible for our study. The exclusion criteria were children with conditions that could make it difficult to complete the questionnaire, such as cerebral palsy, intellectual disability, microcephaly, hemiplegia, and Dunner syndrome. We also excluded children with eating difficulties or problems that could alter food consumption, such as food allergy or intolerance, Asper syndrome, Silver Russell syndrome, Hashimoto’s thyroid, diabetes, kidney disease, Ehler-Danlos, G6PD deficiency, irritable intestine syndrome, megacolon, adrenal hyperplasia, high ferritin, gastritis, and reflux. After data collection, we applied the inclusion and exclusion criteria to the database.

### Study procedures

Our questionnaire was developed in Google Forms. Participants (parents and children) were recruited using the snowball sampling technique [26]. We selected all parents who had access to the questionnaire for the research. The access link to the questionnaire was published on the researchers’ social networks and sent by e-mail to state and municipal education departments, public and private schools, unions of education professionals, nutritionists’ councils, and councils of secretaries of education. The link was available from February to November 2022, when the minimum calculated sample was reached.

Initially, the questionnaire was filled in by the children’s parents, with information about the child’s identification and health. Afterward, the child completed a questionnaire by her/himself. The questionnaire guided the parents to avoid interfering with the children’s responses. The questionnaire was tested in a pilot study. The Dietary Guidelines for the Brazilian Population [9] was made available at the end of the questionnaire. In addition, participants received individual results and a booklet with guidelines on healthy eating, physical activity, and mental health in children written by the researchers. The schools that supported the dissemination of the study received collaboration certificates.

### Measures

The first part of the questionnaire, filled in by the child’s parent, asked for information about the child, such as date of birth, location of residence, the type of school (public or private) that the child attends, and data about the child’s health (for exclusion criterion).

After the parent filled in the first part of the questionnaire, a recommendation was presented for the child to continue completing the form by her/himself. The child was asked to mark the meals he/she did yesterday and answer the Illustrated Questionnaire on Food Consumption for Brazilian Schoolchildren (QUACEB–*Questionário Ilustrado de Consumo Alimentar para Crianças Escolares Brasileiras*) and the Illustrated Questionnaire on Eating and Sedentary Behaviors (QUICAS–*Questionário Ilustrado de Comportamentos alimentares e sedentários*). The QUACEB is a validated questionnaire to investigate the food consumption of elementary schoolchildren between six and ten years of age. This questionnaire is a self-reported recall with 43 foods illustrated [27]. The QUICAS is a validated questionnaire to investigate the eating and sedentary behaviors on the previous day in schoolchildren seven to ten years old. It is an illustrated questionnaire with ten eating behaviors (referring to the act of eating without distractions, with company, on a regular basis, and participation in tasks involved in meal preparation) and five sedentary behaviors (related to the use of television, computer, tablet, cell phone, and video game) with four frequency options each (morning, afternoon, night, and never) [28].

### Data analysis

Regarding food consumption obtained by the QUACEB application, we calculated the NOVA score and the dietary diversity score. The NOVA score was calculated from the sum of reported groups of ultra-processed foods: for each “yes” as an answer, the value 1 was assigned, and for the “no” answers, the value 0 was assigned. Therefore, the score ranged from 0 (none of the foods was consumed on the previous day) to 10 (at least one ultra-processed food from each of the 10 groups was consumed on the previous day) [29]. The same grouping of ultra-processed foods adopted in the original simplified instrument [30] was used in our study (Table 1). For the dietary diversity score, the list of foods adapted for the Brazilian population was used [30], based on the FAO indicator [31]. For the classification of the dietary diversity score, the value 1 was assigned for the presence of each of the 10 food groups: grains and tubers; legumes; meats; eggs; milk; dark green leafy vegetables; fruits and vegetables rich in vitamin A; light green leafy vegetables; other vegetables and other fruits (Table 2). This score was summed up, ranging from 0 to 10.

**Table 2:** Food groups for calculating the NOVA score and the dietary diversity score. Brazil, 2022.

Score	Ultra-processed food groups	Natural or staple food groups
1	Soda	Rice, potato, or cassava/manioc
2	Industrialized juices in cartons	Beans
3	Chocolate milk or flavored yogurt	Beef, pork, chicken, fish, or shrimp
4	Packaged bread	Egg
5	Packaged Salty snacks or crackers	Milk
6	Cookie or packaged sweet cake	Broccoli or kale
7	Chocolate, ice cream, gelatin, or candy	Squash, carrot, papaya, or mango
8	Salami, sausage, baloney, or ham	Lettuce or cabbage
9	Margarine, mayonnaise, or ketchup	Tomato, chayote, or cucumber
10	Instant noodles, frozen lasagna, or pizza	Banana, apple, orange, tangerine, grape, or avocado

Compiled by authors.

The scores were divided into quartiles. The highest risk group was the combination of high consumption of ultra-processed foods (fourth quartile) and low dietary diversity (first quartile).

The sedentary behaviors were classified as “acceptable screen” use behavior for those who reported using television, computer, tablet, cell phone, and video game in a single period (morning or afternoon, or night) or had not used them on the previous day; and “excessive screen” use behavior for those who reported using them (even if it was only one of the screens asked) in two or more periods in the previous day.

A descriptive analysis was performed. For categorical variables, the prevalence was calculated with a Confidence Interval (CI) of 95%. The Poisson regression model with robust variance estimation was performed to calculate the prevalence ratios (PRs) of behavioral variables associated with high consumption of ultra-processed food and low dietary diversity. Eating and sedentary behaviors were considered exposure variables, and food consumption on the previous day in quartiles was the outcome variable. Analyses were conducted both individually and in combination with exposure and outcome variables. A significance level of less 0.05 was considered. The association was adjusted for the variables of the Brazilian macro-region, type of school (proxy of socioeconomic status), gender, and age of the child. Analyses were performed using Stata software version 16.

## Results

The questionnaire was answered by 2,206 people. Of these, 185 were excluded, including 25 responses whose parents had not agreed with the informed consent form; 2 duplicate responses from the same person; 13 children that had not agreed with the informed consent form; 78 children under 6 years and over 11 years old; 10 children with cognitive disorders; and 57 children with eating disorders.

Therefore, the study included 2,021 Brazilian children. According to the last National School Census [25], participants were distributed proportionally to Brazilian macro-regions and type of school (Table 3).

**Table 3:** Participants distribution according to the Brazilian macro-region and type of school. Brazil, 2022.

Variables	Study sample n	Distribution of the study sample %	Adequacy with calculated sample %
**Brazil**	2,021	100.0	100
**Macro-Regions**			
North	294	14.6	130
Northeast	504	24.9	88
Central-West	212	10.5	130
Southeast	697	34.5	88
South	314	15.5	115
**Type of School**			
Public	1,757	86.9	106
Private	264	13.1	73

Compiled by authors.

Most children were male (51.7%), and the mean age was 8 years old (Standard Deviation–SD = 1.5). The mean consumption of ultra-processed foods (NOVA score) on the previous day was approximately 3.5 groups (SD = 2.1) and the mean consumption of natural or staple foods (dietary diversity score) was 5.1 groups (SD = 2.1). Children in the last quartile of the NOVA score (27.6%) consumed five or more groups of ultra-processed foods, and children in the first quartile of the dietary diversity score (39.0%) consumed four or fewer groups of natural or staple foods. Regarding sedentary behavior, the majority of children (75.7%) used screens (such as tablets, cell phones, computers, televisions, or video games) in more than one period (morning, afternoon, or night) on the previous day. In general, children showed healthy eating behaviors but had a high prevalence of eating meals watching TV or using a cell phone (47.2%) and low participation in household activities involving meal preparation (34.2%) (Table 4). On average, children consumed four meals (SD = 1.0) on the previous day.

**Table 4:** Descriptive analysis of food consumption, sedentary and eating behaviors of Brazilian schoolchildren (n = 2,021). Brazil, 2022.

Study variables	Prevalence		
	n	%	CI_95%_
**Food consumption**			
NOVA score^1^			
1^st^, 2^nd^, and 3^rd^ quartiles	1464	72.4	70.4–74.3
4^th^ quartile	557	27.6	25.6–29.5
Dietary diversity score^2^			
1^st^ quartile	789	39.0	36.9–41.2
2^nd^, 3^rd^, and 4^th^ quartiles	1232	61.0	58.8–63.0
NOVA score and dietary diversity score^3^			
NOVA score of1^st^, 2^nd^, 3^rd^ quartiles and diversity score of 2^nd^, 3^rd^, 4^th^ quartiles	1824	90.3	88.9–91.5
4^th^ quartile of NOVA score and 1^st^ quartile of the dietary diversity score	197	9.7	8.5–11.0
**Sedentary behavior**			
Screen time			
Acceptable	492	24.3	22.5–26.3
Excessive	1529	75.7	73.7–77.5
**Eating behavior**			
Eating main meals with distractions			
Without watching television and using cell phones	1067	52.8	50.6–54.9
Watching TV or using a cell phone	954	47.2	45.0–49.4
Eating main meals in company			
With company	1898	93.9	92.8–94.9
Alone	123	6.1	5.1–7.2
Eating main meals at regular times			
At the usual time	1724	85.3	83.7–86.8
At a different time than usual	297	14.7	13.2–16.3
Participation in household activities involving meal preparation			
Yes	691	34.2	32.1–36.3
No	1330	65.8	63.7–67.8
Consumed all three main meals on the previous day			
Yes	1552	76.8	74.9–78.6
No	468	23.2	21.4–25.0
Consumed breakfast on the previous day			
Yes	1736	85.9	84.3–87.4
No	284	14.1	12.7–15.6

Legend: n: Sample; %: Percentage; CI_95%_: 95% confidence interval.

* Sample proportional to the population, with a margin of 30%, according to the last National School Census (INEP, 2022).

^1^ The 1^st^, 2^nd^, and 3^rd^ quartiles represent the consumption of four or fewer ultra-processed foods; and the 4th quartile refers to the consumption of five or more ultra-processed foods.

^2^ The 1st quartile represents the consumption of four or fewer natural or staple foods; and the 2^nd^, 3^rd^, and 4^th^ represent the consumption of five or more natural or staple foods.

^3^ The 4^th^ quartile of the NOVA score and the 1^st^ quartile of the dietary diversity score represent children who consumed five or more ultra-processed foods and four or fewer natural or staple foods; and the group in the 1^st^, 2^nd^, and 3^rd^ quartiles of the NOVA score and the 2^nd^, 3^rd^, and 4^th^ quartiles of the diversity score represent those who consumed less than five ultra-processed foods and more than four natural or staple foods.

Compiled by authors.

The prevalence of food consumption, according to the quartiles of the NOVA score and the dietary diversity score, by eating and sedentary behaviors are presented in Table 5.

**Table 5:** Prevalence of eating and sedentary behaviors based on food consumption the previous day, assessed using NOVA score and dietary diversity score divided into quartiles (n = 2,021). Brazil, 2022.

Exposure variables	4^th^ quartile of the NOVA score^1^ (n = 557)			1^st^ quartile of dietary diversity score^2^ (n = 789)			4^th^ quartile of the NOVA score and 1st quartile of the dietary diversity score^3^ (n = 197)		
	n	%	CI_95%_	n	%	CI_95%_	n	%	CI_95%_
Sedentary behavior: screen time									
Acceptable	85	15.2	12.5–18.5	158	20.0	17.4–22.9	25	12.7	8.7–18.1
Excessive	472	84.8	81.5–87.5	631	80.0	77.0–82.6	172	87.3	81.8–91.2
Eating behavior: eating with distractions									
Without watching television and using cell phones	259	46.5	42.4–50.6	354	44.9	41.4–48.4	78	39.6	32.9–46.6
Watching TV or using a cell phone	298	53.5	49.3–57.6	435	55.1	51.6–58.6	119	60.4	53.4–67.0
Eating behavior: eating in company									
With company	516	92.6	90.2–94.5	730	92.5	90.5–94.2	178	90.4	85.3–93.7
Alone	41	7.4	5.4–9.8	59	7.5	5.8–9.5	19	9.6	6.2–14.6
Eating behavior: eating at regular times									
At the usual time	445	79.9	76.4–83.0	627	79.50	76.5–82.1	147	74.6	68.0–80.2
At a different time than usual	112	20.1	16.9–23.6	162	20.5	17.8–23.5	50	25.4	19.7–31.9
Eating behavior: participation in household activities involving meal preparation									
No	378	67.9	63.8–71.6	564	71.5	68.2–74.5	147	74.6	68.0–80.2
Yes	179	32.1	28.4–36.1	225	28.5	25.5–31.7	50	25.4	19.7–31.9
Eating behavior: consumed all three main meals on the previous day									
No	129	23.2	19.8–26.8	264	33.5	30.3–36.8	69	35.0	28.6–41.9
Yes	428	76.8	73.1–80.1	524	66.5	63.1–69.7	128	65.0	58.06–71.3
Eating behavior: consumed breakfast on the previous day									
No	74	13.3	10.7–16.4	169	21.4	18.7–24.4	39	19.8	14.8–25.9
Yes	483	86.7	83.6–89.3	619	78.6	75.6–81.3	155	80.2	74.0–85.2

Legend: n: Sample; %: Percentage; CI_95%_: 95% confidence interval.

^1^ The consumption of five or more ultra-processed foods.

^2^ The consumption of four or fewer natural or staple foods.

^3^ The consumption of five or more ultra-processed foods and the consumption of four or fewer natural or staple foods.

Compiled by authors.

High consumption of ultra-processed foods and low dietary diversity (4^th^ quartile of the NOVA score and 1^st^ quartile of the dietary diversity score) were associated with sedentary behavior of the screen use [Prevalence Ratio–PR = 2.21; p<0.01]; and with eating behaviors, such as eating while watching television or using a cell phone [PR = 1.71; p<0.01], eating alone [PR = 1.65; p = 0.02], eating at irregular times [PR = 1.97; p<0.01], but not participating in household activities involving meal preparation [PR = 1.53; p<0.01], consuming three main meals [PR = 0.56; p<0.01], and eating breakfast [PR = 0.67; p = 0.01] (Table 6).

**Table 6:** A crude analysis of the association between eating and sedentary behaviors (exposure variables) and food consumption on the previous day (outcome variable), using the NOVA score and food diversity score by Brazilian schoolchildren (n = 2,021). Brazil, 2022.

Exposure variables	4^th^ quartile of the NOVA score^1^ (n = 557)			1^st^ quartile of dietary diversity score^2^ (n = 789)			4th quartile of the NOVA score and 1st quartile of the dietary diversity score^3^ (n = 197)		
	PR	CI_95%_	P value	PR	CI_95%_	P value	PR	CI_95%_	P value
Sedentary behavior: screen time									
Acceptable	1.00			1.00			1.00		
Excessive	1.79	1.4–2.2	<0.01*	1.28	1.1–1.5	<0.01*	2.21	1.5–3.3	<0.01*
Eating behavior: eating with distractions									
Without watching television and using cell phones	1.00			1.00			1.00		
Watching TV or using a cell phone	1.27	1.1–1.5	<0.01*	1.37	1.2–1.5	<0.01*	1.71	1.3–2.3	<0.01*
Eating behavior: eating in company									
With company	1.00			1.00			1.00		
Alone	1.23	0.9–1.6	0.125*	1.25	1.0–1.5	0.02*	1.65	1.0–2.5	0.02*
Eating behavior: eating at regular times									
At the usual time	1.00			1.00			1.00		
At a different time than usual	1.46	1.2–1.7	<0.01*	1.49	1.3–1.7	<0.01*	1.97	1.5–2.6	<0.01*
Eating behavior: participation in household activities involving meal preparation									
No	1.00			1.30	1.1–1.5	<0.01*	1.53	1.1–2.1	<0.01*
Yes	1.09	0.9–1.3	0.23	1.00			1.00		
Eating behavior: consumed all three main meals on the previous day									
No	1.00			1.00			1.00		
Yes	1.00	0.8–1.2	0.99	0.60	0.5–0.6	<0.01*	0.56	0.4–0.7	<0.01*
Eating behavior: consumed breakfast on the previous day									
No	1.00			1.00			1.00		
Yes	1.07	0.8–1.3	0.54	0.60	0.5–0.7	<0.01*	0.67	0.5–0.9	0.01*

Legend: PR: Prevalence ratio; CI _95%_: 95% confidence interval.

*p<0.20.

^1^ The consumption of five or more groups of ultra-processed foods.

^2^ The consumption of four or fewer groups of natural or staple foods.

^3^ The consumption of five or more groups of ultra-processed foods and the consumption of four or fewer groups of natural or staple foods.

Compiled by authors.

The association between eating and sedentary behaviors and food consumption shown in Table 6 (crude analysis) was maintained after adjusting for the variables: type of school, macro-regions, gender, and age of the child (Table 7). Therefore, children who used screens excessively had a 115% greater chance of belonging to the higher risk group (higher consumption of ultra-processed foods and lower consumption of natural or staple foods) than those with acceptably used screens. Regarding eating behaviors, children who presented unhealthy behaviors, such as watching television or using cell phones during meals, eating alone, not eating at regular times, and not participating in household activities involving meal preparation, had 65%, 66%, 91%, and 53% greater chance, respectively, of belonging to the higher risk group (high consumption of ultra-processed foods and low consumption of natural or staple foods) than children who reported healthy eating behaviors on the previous day. Furthermore, children who consumed breakfast and consumed three main meals (breakfast, lunch, and dinner) had a lower chance for each of belonging to the group with high consumption of ultra-processed foods and low consumption of natural or staple foods (Table 7).

**Table 7:** Individual Analysis adjusted for type of school, macro-region, gender, and age of the child for the association between eating and sedentary behaviors (exposure variables) and food consumption on the previous day (outcome variable), using the NOVA score and food diversity score by Brazilian schoolchildren (n = 2,021). Brazil, 2022.

Exposure variables	4^th^ quartile of the NOVA score^1^ (n = 557)			1^st^ quartile of dietary diversity score^2^ (n = 789)			4^th^ quartile of the NOVA score and 1st quartile of the dietary diversity score^3^ (n = 197)		
	PR	CI_95%_	P value	PR	CI_95%_	P value	PR	CI_95%_	P value
Sedentary behavior: screen time									
Acceptable	1.00			1.00			1.00		
Excessive	1.76	1.4–2.2	<0.01*	1.24	1.0–1.4	<0.01*	2.15	1.4–3.2	<0.01*
Eating behavior: eating with distractions									
Without watching television and using cell phones	1.00			1.00			1.00		
Watching TV or using a cell phone	1.23	1.0–1.5	<0.01*	1.34	1.2–1.5	<0.01*	1.65	1.2–2.2	<0.01*
Eating behavior: eating in company									
With company	1.00			1.00			1.00		
Alone	1.25	0.9–1.6	0.09	1.21	1.0–1.5	0.04*	1.66	1.0–2.5	0.02*
Eating behavior: eating at regular times									
At the usual time	1.00			1.00			1.00		
At a different time than usual	1.39	1.2–1.6	<0.01*	1.44	1.3–1.6	<0.01*	1.91	1.4–2.6	<0.01*
Eating behavior: participation in household activities involving meal preparation									
No	-	-	-	1.35	1.2–1.5	<0.01*	1.53	1.1–2.1	<0.01*
Yes	-	-	-	1.00			1.00		
Eating behavior: consumed all three main meals on the previous day									
No	-	-	-	1.00			1.00		
Yes	-	-	-	0.60	0.5–0.7	<0.01*	0.56	0.4–0.7	<0.01*
Eating behavior: consumed breakfast on the previous day									
No	-	-	-	1.00			1.00		
Yes	-	-	-	0.62	0.5–0.7	<0.01*	0.67	0.5–0.9	0.01*

Legend: PR: Prevalence ratio; CI _95%_: 95% confidence interval.

Adjustment variables: Brazilian macro-region, type of school (proxy of socioeconomic status), gender, and age of the child.

*p<0.05.

- Variables with a p-value greater than 0.20 in the crude analysis were excluded from the adjusted analysis.

^1^ The consumption of five or more ultra-processed foods.

^2^ The consumption of four or fewer natural or staple foods.

^3^ The consumption of five or more ultra-processed foods and the consumption of four or fewer natural or staple foods.

Compiled by authors.

In the adjusted multiple Poisson regression, the models were found to be statistically significant (p<0.01). This indicates that at least some of the explanatory variables significantly predict the dependent variables. Specifically, using screens (as a proxy for sedentary behavior) [PR = 1.86; p<0.01], eating at irregular times [PR = 1.62; p<0.01], and consuming all three main meals on the previous day [PR = 0.59; p<0.01] maintained a statistically significant predictive effect in children who fell into the 4th quartile of the NOVA score and the 1st quartile of the dietary diversity score (Table 8).

**Table 8:** Combined analysis adjusted for type of school, macro-region, gender, and age of the child for the association between eating and sedentary behaviors (exposure variables) and food consumption on the previous day (outcome variable), using the NOVA score and food diversity score by Brazilian schoolchildren (n = 2,021). Brazil, 2022.

Exposure variables	4^th^ quartile of the NOVA score^1^ (n = 557)			1^st^ quartile of dietary diversity score^2^ (n = 789)			4^th^ quartile of the NOVA score and 1st quartile of the dietary diversity score^3^ (n = 197)		
	PR	CI_95%_	P value	PR	CI_95%_	P value	PR	CI_95%_	P value
Sedentary behavior: screen time									
Acceptable	1.00			1.00			1.00		
Excessive	1.69	1.4–2.1	<0.01*	1.15	0.9–1.3	0.053	1.86	1.2–2.8	<0.01*
Eating behavior: eating with distractions									
Without watching television and using cell phones	1.00			1.00			1.00		
Watching TV or using a cell phone	1.11	0.9–1.3	0.153	1.18	1.0–1.3	<0.01*	1.32	0.9–1.7	0.053
Eating behavior: eating in company									
With company	1.00			1.00			1.00		
Alone	1.11	0.9–1.4	0.419	0.98	0.8–1.2	0.844	1.23	0.8–1.9	0.339
Eating behavior: eating at regular times									
At the usual time	1.00			1.00			1.00		
At a different time than usual	1.33	1.1–1.6	<0.01*	1.31	1.1–1.5	<0.01*	1.62	1.2–2.2	<0.01*
Eating behavior: participation in household activities involving meal preparation									
No	-	-	-	1.23	1.0–1.4	<0.01*	1.29	0.9–1.7	0.102
Yes	-	-	-	1.00			1.00		
Eating behavior: consumed all three main meals on the previous day									
No	-	-	-	1.00			1.00		
Yes	-	-	-	0.71	0.6–0.8	<0.01*	0.59	0.4–0.8	<0.01*
Eating behavior: consumed breakfast on the previous day									
No	-	-	-	1.00			1.00		
Yes	-	-	-	0.86	0.7–1.0	0.078	1.15	0.7–1.7	0.520

Legend: PR: Prevalence ratio; CI _95%_: 95% confidence interval.

Adjustment variables: Brazilian macro-region, type of school (proxy of socioeconomic status), gender, and age of the child.

*p<0.05.

- Variables with a p-value greater than 0.20 in the crude analysis were excluded from the adjusted analysis.

^1^ The consumption of five or more ultra-processed foods.

^2^ The consumption of four or fewer natural or staple foods.

^3^ The consumption of five or more ultra-processed foods and the consumption of four or fewer natural or staple foods.

Compiled by authors.

## Discussion

This is the first Brazilian online study in a nationwide sample that assessed the relationship between intake of ultra-processed and natural or staple foods with eating and sedentary behaviors on schoolchildren. We discovered an association between food consumption and eating and sedentary behaviors, similar to studies with adults [13,14], adolescents [15–19], and early childhood children [22–24].

In this study, approximately 10% of children were in the highest risk group, considered when children reported high consumption of ultra-processed foods (fourth quartile of the NOVA score) at the same time as low dietary diversity (first quartile of dietary diversity score). The last Household Budget Survey (POF), conducted in 2017–2018, indicated a decline in the availability of natural or minimally processed foods, as well as an increase in the percentage of ultra-processed foods in Brazilian homes [32], which may have affected the food consumption of Brazilian children.

This situation may have been aggravated after the COVID-19 pandemic. After the COVID-19 outbreak, there was a trend of decreasing dietary diversity among children in northern China. This could be related to food availability and lifestyle changes, particularly decreased household income [33]. Lockdown measures have had an impact on diet, inducing an increase in snacking, processed food consumption, and sedentary behavior in children [34]. In Brazil, studies with adults showed that during the period of social restriction, the practice of physical activity decreased, the time in front of screens increased, and the intake of ultra-processed foods increased [35]. In addition, there was an upward trend in the consumption of ultra-processed foods in less economically developed regions by people with less education [36]. We could predict similar effects among Brazilian children.

The consumption of ultra-processed foods harms the quality of the diet, in particular by increasing the energy density of the diet and the levels of sugar, saturated fat, and trans-fat while decreasing the levels of fiber and potassium [37]. On the other hand, the consumption of a diversified diet might raise nutrition levels and help prevent undernutrition, obesity, and non-communicable diseases [31]. A diet rich in fat, salt, and sugar and fewer vegetables and fruits was linked to worse cardiovascular risk markers in 9-year-old Portuguese school children from Lisbon [38]. Furthermore, greater diet diversity was negatively associated with airway inflammation among Portuguese children between 7 and 12 years old from Porto [39].

For dietary diversity, we found the mean consumption of natural or staple foods of 5.1 groups (SD = 2.1). In the West Region of Cameroon, the mean dietary diversity score of the pupils between 5 and 15 years of age was worse than 3.43 (SD = 1.02) (score range: 1–10) [40]. These data indicate that Brazilian children had a more diverse diet than children from other regions. Although 27.6% of the children in our study had a high consumption of ultra-processed foods on the previous day (fourth quartile), most Brazilian children (61.0%) also consumed natural or staple foods (second, third, and fourth quartile).

A positive characteristic in Brazil uncovered by the last Family Budget Survey (POF) was that half of the calories purchased by households came from fresh or minimally processed foods, thus indicating a predominance of food consumption patterns based on natural foods and culinary preparations [32]. This may be part of Brazilian food culture and/or a result of public policies, with emphasis on actions based on the Dietary Guideline for the Brazilian Population, whose golden rule recommends basing your diet on natural or minimally processed foods and avoiding ultra-processed foods [9]

Regarding sedentary behavior, excessive use of screens during childhood and adolescence has been associated with different health risks. Higher sedentary screen time was associated with a higher risk of metabolic syndrome in children 6–14 years of age in Beijing [41], with excessive weight in Chinese children and adolescents from 6 to 18 years old [42], and with overweight/obesity in 6–7-year-old Italian children [43]. Similarly, school-age children who spent more than 2 hours per day in sedentary activities or screen-based activities (tablet/computer/mobile phone use, watching TV, and video game use) were associated with unhealthy dietary habits, such as inadequate fruits and vegetables consumption, drinking sugary beverages daily, skipping breakfast, consuming carbonated drinks, consuming food in front of the television, and intake of at least one ultra-processed food per day [43–46].

Regarding eating behavior, we noted that approximately half of the children (47.2%) ate main meals (lunch or dinner) with distractions, watching TV, or using a cell phone. Similarly, the SISVAN indicates that 54% of children between 5 and 9 years old monitored in primary healthcare habitually eat their meals watching television [7]. Cell phones on the table and television sets turned on while eating can negatively affect health, with problems such as overeating (less attention to their food and consuming larger portions), poor digestion (chewing food less often, which can make it harder for their bodies to digest the food properly), and poor nutritional intake (choosing junk food or snacks that are high in calories, fat, and sugar, and less likely to eat fruits and vegetables) [9].

A positive finding of our study was that most children (93.9%) reported eating the main meals in company. Eating in company is recommended by the Dietary Guidelines for the Brazilian Population because meals eaten in company avoid eating quickly and favor more appropriate eating environments [9]. This warns against substituting consumption of traditional meals (seated at a table, with a plate, tableware, and with family or friends around) for ultra-processed foods that can be eaten while carrying out other activities (study/work) and in any space (desk, means of transport). Thus, it aims to reestablish the normative systems of dietary practices, which are considered protective against the consumption of ultra-processed foods [47]. In adolescents, eating meals with their parents and avoiding sedentary behaviors, such as watching television during meals, were found to be significantly associated with healthy behavior towards food consumption, this highlights the influence of the environment on individual choices [16,19].

Furthermore, most of the children in this study (85.3%) reported eating main meals at the usual and regular times. Beyond the quality of food, when we eat also plays a key role in health. The timing of the meals of the day could impact metabolism, glucose tolerance, obesity-related factors, and the circadian system. Therefore, eating at regular times could be an effective dietary strategy to prevent obesity, type 2 diabetes, and cardiovascular disease [48,49].

In contrast, a minority of children (34.2%) report participation in household activities involving meal preparation. Having culinary skills and the pleasure of cooking can reduce the consumption of ultra-processed foods, increase the consumption of fruits and vegetables, and reduce the risk of being overweight and obese [50]. A review of studies demonstrated improvement in children’s psychosocial outcomes, nutrition behavior, and food consumption after participating in hands-on meal preparation activities [51]. Another review identified improved overall dietary quality, increased consumption of fruits and vegetables, greater preference for vegetables, higher self-efficacy for cooking, and choosing healthy foods after involvement in-home meal preparation [52].

On the other hand, most of the children participating in this study (76.8%) consumed all three main meals (breakfast, lunch, and dinner) on the previous day. That is much more than observed by SISVAN, in which 16% of children between 5 and 9 years old had the habit of eating at least the three main meals [7]. This discrepancy may have been caused by the socioeconomic differences of the evaluated public. The Food Guide for the Brazilian Population recommends that individuals should consume three main meals daily. This is important to meet their nutritional requirements and to maintain a balanced and healthy diet [9]. Finnish children, ages 6–8, who ate three meals a day had smaller waist circumferences and a 63% lower risk of being overweight or obese than those who skipped some major meals [53]. Adolescents who had the habit of having three main meals a day (PR = 0.81; 95% CI 0.73;0.89 p < 0.05) and who consumed fresh fruit the previous day (PR = 0.91; 95% CI 0.84;0.98 p < 0.001) had a lower prevalence of obesity [54].

In addition, it was observed that 85.9% of schoolchildren consumed breakfast on the previous day. This result is similar to the findings of studies conducted in two different places in Brazil: the city of Florianopolis, where breakfast consumption was reported by 85% of the children with 7 to 13 years, and the state of Minas Gerais, where breakfast was consumed by 79.9% of children aged 8 and 9 years [55,56]. Breakfast consumption was inversely associated with ultra-processed dietary patterns [55]. Furthermore, skipping breakfast was associated with a more proinflammatory diet in school-age children, and there was significant interaction with sedentary behavior [56], reinforcing the importance of this meal in this stage of life.

We found that schoolchildren with unhealthy eating and sedentary behaviors (with excessive screen time use, with screens during meals, eating alone, not eating at regular times, not participating in household activities involving meal preparation, not consuming three main meals, or skipping breakfast) had a greater chance of belonging to the higher risk group (higher consumption of ultra-processed foods and lower consumption of natural or staple foods). This risk was identified regardless of the type of school, the macro-region, gender, or age of the child, and reached 91% and 115% for behaviors of eating at irregular times and for excessive screen use, respectively, showing the relevance of these behaviors on the quality of the diet in this stage of life, as described before. These results suggest that interventions aimed at promoting child health should not only focus on diet quality but also take behaviors into consideration.

Our study has some limitations. One of them is the use of a non-probabilistic sample; nonetheless, this study includes a sizable population proportionally from five geographic regions in Brazil. Another limitation includes data collected using a parent-guided online survey, although the study used validated tools and a pilot test was conducted before use among this population. In addition, the cross-sectional design of the study prevents the inference of causality in the observed associations; however, the cross-sectional design is a starting point for future longitudinal studies that will be able to perform more robust causal analyses. Finally, we tested associations considering only the previous day of food consumption. It would have been useful to consider the habit or eating frequency, as it may have been an atypical day in the child’s diet. However, this kind of questionnaire could not be filled in by the children themselves, because of their inability to correctly report the frequency of a complex behavior such as eating.

## Conclusions

In conclusion, sedentary and unhealthy eating behaviors were associated with the consumption of ultra-processed foods and low dietary diversity in Brazilian schoolchildren. We suggest prioritizing strategies aimed at accessing a greater diversity of food groups and less ultra-processed foods. Additionally, we recommend that future studies focus on evaluating children at lower nutritional risk (those with low consumption of ultra-processed foods and high dietary diversity) to further understand the dynamics of their eating and sedentary behaviors. Furthermore, interventions for promoting healthy eating behaviors, such as eating without distractions from television or cell phones, eating in the company of others, adhering to regular mealtimes, and participating in activities involving meal preparation while discouraging screen use, may be useful to prevent negative consequences. This implies that interventions in clinical practice and public health policies should take a holistic approach to children’s health. This includes considering not only the quality of food consumption but also their eating and sedentary behaviors.

## Supporting information

S1 Appendix Data set. (XLSX)
